# Experiences of Assisted Living Communities Affected by Hurricane Irma: Leadership, Lessons Learned

**DOI:** 10.1093/geroni/igaa057.2611

**Published:** 2020-12-16

**Authors:** Kathryn Hyer, Lindsay Peterson, David Dosa, Joseph June, Debra Dobbs

**Affiliations:** 1 University of South Florida, Tampa, Florida, United States; 2 Brown University, Barrington, Rhode Island, United States; 3 School of Aging Studies, University of South Florida, Tampa, Florida, United States

## Abstract

Little is known about the effects of disasters on assisted living community (ALC) residents. This is a concern given the growth of the AL industry and the increasing numbers of AL residents with functional limitations and chronic health conditions. This research examined the experiences of AL administrative staff to better understand the impact of Hurricane Irma. Qualitative interviews were conducted with representatives of ALCs across Florida (N=70), and transcripts were analyzed using Atlas.ti version 8. Research team members met regularly to reach consensus on codes, identifying five major themes across the interviews, 1) planning and preparation, 2) leadership, including plan execution and managing the unexpected, 3) effects/consequences of the storm, including effect on residents and staff, 4) lessons learned, and 5) electrical power. Results provide a broad view of ALC preparedness, how it varies across different types of ALCs and implications for resident wellbeing and future emergency planning.

